# The Role of Radiotherapy for Patients with Unresectable Locally Advanced Breast Cancer following Neoadjuvant Systemic Therapy

**DOI:** 10.1155/2023/5101078

**Published:** 2023-02-17

**Authors:** Xiaofang Wang, Jin Meng, Xiaomeng Zhang, Li Zhang, Xingxing Chen, Zhaozhi Yang, Xin Mei, Xiaoli Yu, Zhen Zhang, Zhimin Shao, Guangyu Liu, Xiaomao Guo, Jinli Ma

**Affiliations:** ^1^Department of Radiation Oncology, Fudan University Shanghai Cancer Center, Shanghai 200032, China; ^2^Department of Oncology, Shanghai Medical College, Fudan University, Shanghai 200032, China; ^3^Shanghai Clinical Research Center for Radiation Oncology, Shanghai 200032, China; ^4^Shanghai Key Laboratory of Radiation Oncology, Shanghai 200032, China; ^5^Department of Breast Surgery, Fudan University Shanghai Cancer Center, Shanghai 200032, China

## Abstract

**Background:**

For locally advanced breast cancer (LABC) patients who remained unresectable after neoadjuvant systemic therapy (NST), radiotherapy (RT) is considered as an approach for tumor downstaging. In this study, we attempted to discuss the value of RT for patients with unresectable or progressive disease in the breast and/or regional nodes following NST.

**Methods:**

Between January 2013 and November 2020, the data for 71 patients with chemo-refractory LABC or de novo bone-only metastasis stage IV BC who received locoregional RT with or without surgical resection were retrospectively analyzed. Factors associated with tumor complete response (CR) were recognized using logistic regression. Locoregional progression-free survival (LRPFS) and progression-free survival (PFS) were calculated using the Kaplan–Meier method. The Cox regression model was applied to recognize the recurrence risk factors.

**Results:**

After RT, 11 patients (15.5%) achieved total cCR. Triple-negative subtype (TNBC) was associated with a lower total cCR rate compared with other subtypes (*p* = 0.033). 26 patients proceeded to surgery, and the operability rate was 36.6%. 1-year LRPFS and PFS were 79.0% and 58.0%, respectively, for the entire cohort. Surgical cases had an improved 1-year LRPFS (*p* = 0.015), but not 1-year PFS (*p* = 0.057), compared with definitive RT cases. Non-any cCR was the most prominent predictor of a shorter LRPFS (*p* < 0.001) and PFS (*p* = 0.002) in the multivariate analysis. Higher TNM stage showed a trend toward a shorter LRPFS time (*p* = 0.058), and TNBC (*p* = 0.061) showed a trend toward a shorter PFS interval.

**Conclusions:**

This study demonstrated that RT was an effective tumor downstaging option for chemo-refractory LABC. For patients with favorable tumor regression, surgery following RT might bring survival benefits.

## 1. Introduction

Locally advanced breast cancer (LABC) is a subset of invasive breast cancer, constituting 5–10% of newly diagnosed cases. LABC describes the advanced status of lesions in the breast and/or regional nodes with the evaluation of clinical examination and radiographic methods. In the 8^th^ AJCC clinical staging system [1], LABC can be divided into (1) operable diseases (T3N0-1) with breast tumors larger than 5 cm; and (2) inoperable diseases (T4/N2-3) with chest wall/skin invasion, inflammatory breast cancer (IBC), with or without fixed or very bulky axillary nodal disease, and/or supraclavicular (SCV)/infraclavicular (ICV)/internal mammary nodal (IMN) involvement. Neoadjuvant systemic therapy (NST) is the current recommendation for the initial treatment of LABC to render inoperable tumors resectable [2]. However, up to one-third of LABCs remain unresectable due to poor response to systemic therapy. For this subset of patients, radiotherapy (RT) represents another option with the aim of downstaging. In addition, among de novo stage IV breast cancer patients, those with bone-only metastasis were demonstrated to have better prognosis and might benefit from local therapy following NST [3, 4]. In this study, we attempt to discuss the value of RT in LABC patients with inoperable or progressive disease after NST.

## 2. Materials and Methods

### 2.1. Patients

Of all the patients diagnosed with breast cancer and treated with RT at Fudan University Shanghai Cancer Center between January 2013 and November 2020, 110 patients were identified with locally advanced diseases that could not be rendered resectable by systemic treatment. Patients who had metastatic diseases other than bone metastasis (*n* = 25), or with occult breast cancer (*n* = 7), or lacking evaluation post-RT (*n* = 5), or received local excision of primary tumor with no residual disease before RT (*n* = 2) were excluded. The final cohort included 71 patients for analysis. The review of data was approved by the Ethical Committee and Institutional Review Board of our center.

Patient characteristics including age at diagnosis, menopausal status, laterality, tumor histopathology, the status of the primary tumor (T)/regional lymph nodes (N)/distant metastasis (M), site of regional nodes, Ki-67 value, and estrogen receptor (ER)/progesterone receptor (PR)/human epidermal receptor 2 (HER2) status were extracted from the medical records. The TNM stage was classified according to the AJCC Cancer Staging Manual, 8th Edition. Pre-/post-NST/RT clinical tumor stage (c) was generally determined based on physical examination, imaging studies, such as chest computed tomography (CT), breast magnetic resonance imaging (MRI), bone emission computed tomography (ECT), and abdominal ultrasound, and clinical notes documented by patient's oncologists. To reflect tumor response to treatment more clearly, this study allowed T4 or N3 tumors downstaged with tumor regression after NST/RT. Pathological stage (*p*) was determined according to pathological report after surgery. The expression of biomarkers (ER, PR, HER2, and Ki-67) was measured by immunohistochemistry (IHC). ER and PR were defined as positive with the value ≥ 1% [5]. HER2 was considered positive if 3+ staining, negative if 0 or 1+ staining, and equivocal if 2+ staining on IHC. When IHC was equivocal, tumors were considered HER2 positive with amplification (ratio ≥ 2.0) by fluorescence in situ hybridization (FISH) analysis [6]. Hormonal receptor (HR)+ was defined as ER+ and/or PR+, and HR− was defined as both ER− and PR−. Luminal A was defined as HR+/HER2− and Ki-67 < 30%, HER2-overexpressed was defined as HR−/HER2+, triple-negative (TNBC) was defined as HR−/HER2−, and others were classified in Luminal B subtype.

### 2.2. Treatment

All patients underwent NST as the initial treatment according to the international guidelines. RT was administered as the salvage treatment for unresectable diseases after NST based on institutional multidisciplinary discussion. Generally, a prescription dose of 50 Gy in 25 fractions was delivered to the breast and the regional nodes, which included axillary (ALN), SCV, and ICV with or without IMN using the simplified inverse-planningintensity-modulated RT (IMRT) technique. An additional boost was sequentially delivered to the gross tumor at the discretion of treating physician. During RT, concurrent chemotherapy was used in selected patients. Following RT, mastectomy (MRM) with flap repair or chest wall reconstruction if in need, breast-conserving surgery (BCS) in selected patients, and axillary lymph node dissection (ALND) were conducted according to tumor response, surgeon's assessment, and patient's willingness.

### 2.3. Endpoints

Clinical response in the breast and regional nodes was defined according to RECIST criteria [7]: no palpable/visible abnormality at the site indicated a clinical complete response (cCR). Pathological CR (pCR) is defined as no invasive cancer in the breast and ALNs at surgery. We defined breast-only CR, nodal-only CR, or both CR as any CR, distinguishing from breast and nodal total CR. Failing to achieve any CR was considered non-any CR. Operability rate was defined as the proportion of patients who proceeded to surgery after NST and RT. Locoregional progression (LRP) was defined as clinical, radiographic, or pathological evidence of disease progression within ipsilateral breast/chest wall (CW) or regional nodes (i.e., ipsilateral ALN, SCV, ICV, or IMN). Progression at sites other than locoregional was considered as distant progression (DP). Locoregional progression-free survival (LRPFS) was measured from the date of completion of RT to the time of LRP or the last visit. Progression-free survival (PFS) was defined as the interval from the date of completion of RT to LRP, DP, or the last visit.

### 2.4. Statistical Analysis

Patient characteristics between groups were compared using Pearson's *χ*^2^ test. Factors associated with tumor CR were recognized using the logistic regression in the multivariate analyses. Estimates of LRPFS and PFS rates were calculated using the Kaplan–Meier method and compared between groups with the log-rank test. Factors associated with LRPFS and PFS were recognized using the Cox regression model in the univariate and multivariate analyses. The level of significance was set at *p* < 0.05 (two-sided), using SPSS 26.0.

## 3. Results

### 3.1. Patient and Treatment Characteristics

The median age was 50 years (range, 26–74 years) for the entire cohort. The primary tumor stage at diagnosis was cT1-2 in 15.5% of patients (*n* = 11) and cT3-4 in 84.5% of patients (*n* = 60), including 27 cases with IBC. The nodal stage at diagnosis was cN0-1 in 23.9% (*n* = 17), cN2 in 23.9% (*n* = 17), and cN3 in 52.1% of patients (*n* = 37). The most common site of positive regional nodes was ALN alone (*n* = 30; 42.3%), followed by ALN + SCV (*n* = 25; 35.2%) and ALN + IMN (*n* = 9; 12.7%). Stages cIIIB and cIIIC were common, corresponding to 38.0% and 45.1% of cases, respectively. Stage IV patients with bone-only metastasis accounted for 12.7% (*n* = 9) of all cases. On IHC staining, the percentage of Luminal A, Luminal B, HER2-overexpressed, and triple-negative subtype was 14.1%, 21.1%, 21.1%, and 43.7%, respectively (Table 1).

At least two cycles of chemotherapy were administered prior to RT in 70 cases, and the other patient received neoadjuvant endocrine therapy because of serious medical complications. 95.2% (*n* = 20) of HER-positive patients received anti-HER2 therapy before RT.

A total of 45 patients (63.4%) received definitive RT (DRT) alone, and 26 patients (36.6%) proceeded to surgical resection after neoadjuvant radiotherapy (NART). Table 1 compares the patient characteristics at time of primary diagnosis, and Table 2 summarizes treatment-related characteristics between DRT and NART subgroups. All patients received external beam RT to the breast and regional nodes (ALN, ICV/SCV, with or without IMN), with a median dose of 50 Gy (range, 30–56 Gy) in 25 fractions (range, 10–28 fractions). An additional boost to the gross tumor in the breast and/or regional nodes was sequentially administered in 43 patients (60.6%), including 35 patients in the DRT group and 8 patients in the NART group.

Concurrent chemotherapy was used in 40 patients (56.3%); the most common regimen was cisplatin (*n* = 19), followed by capecitabine (*n* = 17). None of the stage IV patients received palliative RT to the involved bones. Surgery was conducted in 26 patients; 25 patients received MRM, including 5 patients with flap repair or chest wall reconstruction, and 1 patient underwent BCS and ALND. The operability rate was 36.6% in this cohort. The median duration between RT and surgery was 10 weeks (range, 3–54 weeks).

### 3.2. Tumor Response after RT

The median time from RT completion to clinical assessment of tumor response to RT was 5 weeks (range, 0–22 weeks). 11 patients achieved total cCR, and 31 patients achieved breast-only or nodal-only cCR for the entire cohort. Among the 45 patients who underwent RT alone, 1 (2.2%) achieved breast-only cCR, 17 (37.8%) achieved nodal-only cCR, and 9 (20.0%) achieved total cCR. Of the 26 patients who underwent surgical resection after NART, 1 (3.8%) achieved breast-only cCR, 12 (46.2%) achieved nodal-only cCR, and 2 (7.7%) achieved total cCR. Furthermore, among those who proceeded to surgery, 5 (19.2%) achieved both breast and axillary nodal pCR. The proportion of patients who achieved breast-only or axillary nodal-only pCR was similar to the corresponding cCR rate at the time of clinical evaluation after RT (Table 3).

The correlation of cCR with various prognostic factors is shown in Table 4. In univariate analysis for the entire cohort, molecular subtype was significantly associated with the total cCR (*χ*^2^ = 14.98, *p* = 0.002) and any cCR (*χ*^2^ = 10.90, *p* = 0.012). However, the association of other clinical and treatment-related factors including age, pre-RT TNM stage, radiation boost, and concurrent chemotherapy with total cCR or any cCR was not found to be statistically significant. In the following multivariate analysis, TNBC was demonstrated to be the only predictor of a lower cCR rate in terms of total cCR (adjusted hazard ratio (HR), 0.46; 95% confidence interval (CI), 0.23–0.94; *p* = 0.033) or any cCR (HR, 0.60; 95% CI, 0.42–0.84; *p* = 0.003) (Table 4). Among patients who received surgery after NART, molecular subtype was significantly associated with total pCR as well (*χ*^2^ = 10.23, *p* = 0.017); the pCR rate was higher in luminal subtype and HER2-overexpressed subtype (5/11, 45.5%) than in TNBC subtype (0/15, 0.0%). However, due to the limited sample size and number of events, the results of the multivariate analysis in the NART subgroup were not stable, which were not shown in the paper.

### 3.3. Survival Outcomes

**Figure 1:**
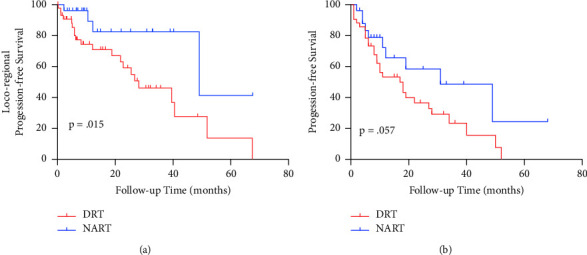
LRPFS (a) and PFS (b) of patients in DRT and NART subgroups. DRT = definitive radiotherapy; NART = neoadjuvant radiotherapy; LRPFS = locoregional progression-free survival; PFS = progression-free survival.

The median follow-up time was 14.8 months from the completion of RT. 26 patients (36.6%) failed to secure locoregional control, and 53.8% of them were TNBC. Overall, the most common recurrent site was breast/CW alone (*n* = 18; 69.2%), followed by regional nodes alone (*n* = 5; 19.2%) (Table 5). 1-year LRPFS was 79.0% for the entire cohort. Statistically significant difference was observed for the 1-year LRPFS (72.7% vs. 89.3%, *p* = 0.015) between DRT and NART subgroups. A total of 36 patients (50.7%) developed DP, including 21 patients with concomitant LRP (Table 5). For the entire cohort, the 1-year PFS was 58.0%. NART subgroup showed a trend toward improvement of 1-year PFS compared with DRT subgroup (72.0% vs. 50.5%, *p* = 0.057) (Figure 1).

Factors associated with LRPFS and PFS are shown in Table 6. In the univariate analysis, older age (*p* = 0.033), TNBC (*p* = 0.006), and non-any cCR (*p* < 0.001) were correlated with a worse LRPFS. TNBC (*p* = 0.003) and non-any cCR (*p* = 0.001) were associated with a shorter PFS. Other factors including menopausal status, TNM stage, radiation boost, and concurrent chemotherapy were not significantly associated with LRPFS. These factors along with age were not significantly associated with PFS as well. In the multivariate analysis, non-any cCR was the most prominent predictor of a shorter LRPFS (HR, 5.65; 95% CI, 2.31–13.85; *p* < 0.001) and PFS (HR, 2.72; 95% CI, 1.44–5.14; *p* = 0.002). Although statistically significant differences were not achieved, higher TNM stage showed a trend toward a shorter LRPFS time (*p* = 0.058), and TNBC (*p* = 0.061) showed a trend toward a shorter PFS interval.

## 4. Discussion

CR has been proved to be a prognostic factor for LABC patients who underwent NST; however, failure in response to systemic therapy is associated with a poor prognosis. For this subgroup of patients, the treatment remains a clinical challenge. RT is considered as a salvage approach for tumor downstaging, and several studies have discussed the value of RT in the multidisciplinary treatment of unresectable LABC [8–10]. Mladenovic et al. assessed tumor response and survival in 134 LABC patients who were treated with NART but without NST and reported total cCR in 21.6% and breast pCR in 15% of patients. Patients who achieved cCR had a longer overall survival (OS) (*p* = 0.038) and a trend toward improvement in DFS compared with patients achieving clinical partial response and stable disease [11]. Sousa et al. published results of 76 LABC patients who received NART, including 57% of patients who were refractory to NST. Among them, 14.5% of patients achieved total pCR, and patients with pathological response (pR) > 90% have a better overall survival (*p* = 0.004) and tend to have a better PFS (*p* = 0.059) [12]. These results indicated that with the administration of NART, CR remained the prognostic factor of survival. In this study, we reported total pCR in 19.2%, breast-only pCR in 3.8%, and axillary nodal-only pCR in 46.2% of patients who proceeded to surgery after RT. For the entire cohort, total cCR was observed in 15.5%, breast-only cCR in 2.8% and nodal-only cCR in 40.8% of LABC patients. Although 63.4% of patients had T4 disease in this study, we showed a similar cCR rate as previous studies (10–29.5%) in LABC after RT [13–17].

Molecular subtype was significantly associated with CR in univariate and multivariate analyses. Total cCR for the entire cohort and total pCR for surgical cases are generally lower in TNBC as compared with other subtypes. In the entire cohort, only 3.2% of TNBC patients achieved total cCR and 38.7% achieved any cCR; a similar tendency of total pCR was observed in patients who received surgery (0.0%). Contrary to our results, Riet et al. reported that triple-negative status was the only predictor of pCR in the multivariate analysis (*p* = 0.002) [16]. Sousa et al. demonstrated that there were no statistical differences between pR and the intrinsic subtypes (*p* = 0.092) [12]. Adams et al. reported that the pathologic response rate was higher in patients with HR-negative tumors than in patients with HR-positive tumors (*p* < 0.0001) [18]. The contradictory results might be due to the following: (1) the heterogeneity of patients: 96.8% of TNBC were staged IIIB-IV, and 44.1% of patients staged IIIB-IV were TNBC in our study; (2) patients in this study were heavily treated with the median of 8 cycles of chemotherapy administered, and tumors might show resistance to concurrent chemotherapy; and (3) TNBC was more aggressive without effective treatment.

Conventionally, surgical resection is a goal to be pursued in LABC by making curative treatment possible. Several studies have reported the operability rate of 18%–82% in patients who remained unresectable after NST and subsequently received NART [19–22]. In this study, 26 out of 71 patients (36.6%) proceeded to surgery after the completion of NART. Generally, mastectomy was the routine surgical approach for patients with LABC. The resection of huge and advanced tumors frequently results in extensive chest wall skin defects, so it is essential for post-mastectomy chest wall reconstruction. However, immediate chest wall reconstruction (ICWR) remains controversial after LABC mastectomy with the consideration that ICWR may delay adjuvant radiation and increase the risk of locoregional recurrence. Several recent studies have explored the feasibility of ICWR. Chang et al. reported that for LABC patients, autologous breast reconstruction could be performed safely in terms of complications regardless of pre-operative or post-operative radiation therapy [23]. In the systemic review of 18 studies, Singh et al. demonstrated that neoadjuvant radiotherapy (NART) and immediate breast reconstruction (IBR) could achieve acceptable post-operative complication (3–36%), favorable cosmetic outcomes (78% by physicians and 89% by patients), and low locoregional recurrence (LRR) (0–10% with the follow-up period of 16.2–96 months) in LABC, indicating the safety of this treatment both technically and oncologically [24]. Song et al. analyzed the survival of 104 LABC cases, who had huge chest wall defects after mastectomy, demonstrating that wide resection followed by ICWR is oncologically safe [25]; moreover, ICWR is also esthetically acceptable in the study, with favorable cosmetic outcomes (83.0%) and relatively high patient satisfaction scores (90.0%). Taken together, LABC patients who received NART might be the proper candidates of ICWR, and randomized studies are still needed for high-quality evidence.

The prognosis of inoperable LABC is poor. Yee et al. reported the median LRPFS of 13.4 months (range, 0–79.8 months) and overall progression-free survival (OPFS) of 11.7 months (range, 0–79.8 months) in a cohort of unresectable LABC with TNBC and metastatic diseases excluded [22]. Woodward et al. included 32 patients who encountered chemo-refractory treatment in a phase 2 study and demonstrated 1-year locoregional recurrence-free survival (LRRFS) of 65% and 1-year OS of 54% with concomitant chemo-radiotherapy; meanwhile, TNBC owed worse 1-year LRRFS compared with luminal and HER2-overexpressed subtypes (20% vs. 63%, *p* = 0.007) [9]. Coelho et al. studied the survival of chemo-refractory LABC patients who were rendered operable after concomitant radio-chemotherapy and reported 2- and 5-year disease-free survival (DFS) of 45.6% and 35.1% and 2- and 5-year OS of 76.7% and 36.4%, respectively [10]. In this heavily treated cohort with 93.0% of patients diagnosed with T4 or N3 and 43.7% with TNBC, we reported 79.0% for 1-year LRPFS and 58.0% for 1-year PFS. Non-any cCR was significantly associated with a poorer LRPFS (*p* < 0.001) and PFS (*p* = 0.002) in the multivariate analysis. In accordance with our data, previous studies reported that CR after NST or NART is a strong predictor of improved survival outcomes [26–28]. Meanwhile, our results showed that surgical resection after NART could achieve a longer LRPFS (*p* = 0.015) and a trend toward improvement of 1-year PFS (*p* = 0.057), and the possible explanation that makes the difference might be due to the following: (1) surgery removed gross tumor and tumor burden was alleviated and (2) patients who received RT alone had more advanced disease and progressive disease compared with surgical cases following RT. From this study, we consider that both definitive RT and surgery after NART are optional for chemo-refractory LABC, while for patients with favorable tumor regression, surgery following NART might bring more survival benefits.

The value of local treatment for de novo metastatic breast cancer is debated [29, 30]. In the subgroup analysis of this study, patients with bone metastasis could achieve similar 1-year LRPFS (*p* = 0.720) and PFS (*p* = 0.892) with non-metastasis cases. In a randomized phase III trial (MF07-01), Soran reported that in patients with bone-only metastases, risk of death was statistically lower in locoregional treatment group (*p* = 0.04) [3]. Although Turanli failed to observe extra survival benefits with local therapy in patients with isolated bone metastasis, he demonstrated that the response to NST is the major factor on survival in the multivariate analysis [31]. Despite the preliminary results from limited number of patients, it might be feasible to consider local treatment in patients with metastasis to bone-only, especially for patients who have favorable response to NST. Further studies with larger cohort of patients are still needed to verify the results.

## 5. Conclusions

This retrospective study from a single institution demonstrated that RT was an effective downstaging option for patients with heavily-treated LABC. Patients with TNBC are correlated with a lower CR rate. Non-any cCR is a predictor of worse LRPFS and PFS after NST and RT, and patients achieving cCR tend to have a longer survival. For patients with favorable tumor regression, surgery after RT might bring survival benefits. With similar NST and local-regional RT applied, patients with de novo bone-only metastasis could achieve similar LRPFS and PFS with non-metastasis cases. Further prospective studies with larger cohort of patients are still needed to verify the value of RT in this clinical scenario.

## Figures and Tables

**Table 1:** Patient characteristics at time of primary diagnosis and comparison between DRT and NART subgroups.

Parameters		Total	DRT (*n* = 45)	NART (*n* = 26)	*p*
		No. (%)	No. (%)	No. (%)	
Median age (range) (years)		50 (26–74)	48 (30–74)	53 (26–72)	

Menopausal status	Pre/peri	38 (53.5)	25 (55.6)	13 (50.0)	0.640
	Post	32 (45.1)	19 (42.2)	13 (50.0)	
	Male	1 (1.4)	1 (2.2)	0 (0.0)	

Laterality	Left	37 (52.1)	25 (55.6)	12 (46.2)	0.445
	Right	34 (47.9)	20 (44.4)	14 (53.8)	

cT	cT1	2 (2.8)	2 (4.4)	0 (0.0)	0.002
	cT2	9 (12.7)	8 (17.8)	1 (3.8)	
	cT3	15 (21.1)	14 (31.1)	1 (3.8)	
	cT4	45 (63.4)	21 (46.7)	24 (92.3)	
	cT4d	27 (38.0)	13 (28.9)	14 (53.8)	

cN	cN0	3 (4.2)	2 (4.4)	1 (3.8)	0.066
	cN1	14 (19.7)	5 (11.1)	9 (34.6)	
	cN2	17 (23.9)	10 (22.2)	7 (26.9)	
	cN3	37 (52.1)	28 (62.2)	9 (34.6)	

Site of regional nodes	None	3 (4.2)	2 (4.4)	1 (3.8)	0.241
	ALN	30 (42.3)	16 (35.6)	14 (53.8)	
	SCV	1 (1.4)	0 (0.0)	1 (3.8)	
	ALN + SCV	25 (35.2)	19 (42.2)	6 (23.1)	
	ALN + IMN	9 (12.7)	5 (11.1)	4 (15.4)	
	ALN + SCV + IMN	3 (4.2)	3 (6.7)	0 (0.0)	

cM	cM0	62 (87.3)	36 (80.0)	26 (100.0)	0.015
	cM1 (bone)	9 (12.7)	9 (20.0)	0 (0.0)	

Clinical TNM stage (pre-treatment)	IIIA	3 (4.2)	3 (6.7)	0 (0.0)	0.001
	IIIB	27 (38.0)	10 (22.2)	17 (65.4)	
	IIIC	32 (45.1)	23 (51.1)	9 (34.6)	
	IV	9 (12.7)	9 (20.0)	0 (0.0)	

Biologic subtype	Luminal A	10 (14.1)	8 (17.8)	2 (7.7)	0.290
	Luminal B	15 (21.1)	11 (24.4)	4 (15.4)	
	HER2-overexpressed	15 (21.1)	10 (22.2)	5 (19.2)	
	Triple-negative	31 (43.7)	16 (35.6)	15 (57.7)	

DRT = definitive radiotherapy; NART = neoadjuvant radiotherapy; ALN = axillary lymph nodes; SCV = supraclavicular; ICV = infraclavicular; IMN = internal mammary nodes.

**Table 2:** Treatment summary of DRT and NART subgroups.

Parameters	Total (*n* = 71)	DRT (*n* = 45)	NART (*n* = 26)	*p*
	No. (%)	No. (%)	No. (%)	
*Neoadjuvant treatment*				
Chemotherapy	70 (98.6)	44 (97.8)	26 (100)	0.444
Median no. of lines of chemotherapy (range)	2 (1–4)	1 (1–4)	2 (1–3)	—
Median no. of cycles of chemotherapy (range)	8 (2–27)	8 (2–27)	7 (2–14)	—
Endocrine therapy	5 (7.0)	4 (8.9)	1 (3.8)	0.424
HER2-targeted therapy	20 (28.2)	13 (28.9)	7 (26.9)	0.859

*Radiation treatment*				
Median dose (Gy) (range)	50 (30–56)	50 (30–56)	50 (46–50)	—
Median no. of fractions (range)	25 (10–28)	25 (15–28)	25 (23–25)	—
Boost to tumor bed/regional nodes	43 (60.6)	35 (77.8)	8 (30.8)	<0.001
Median boost dose (Gy) (range)	14 (6–20)	14 (10–20)	13 (6–20)	—
Median no. of boost fractions (range)	7 (3–10)	7 (5–10)	6.5 (3–10)	—
Fractionation schemes				<0.001
45 Gy/15 Fx	1 (1.4)	1 (2.2)	0 (0.0)
46–50 Gy/23–25 Fx	28 (39.4)	10 (22.2)	18 (69.2)
56–70 Gy/28–35 Fx	42 (59.2)	34 (75.6)	8 (30.8)
Concurrent chemotherapy	40 (56.3)	24 (53.3)	16 (61.5)	0.502
Cisplatin	19 (26.8)	9 (20.0)	10 (38.5)	0.197
Capecitabine	17 (23.9)	11 (24.4)	6 (23.1)
Taxane-based	4 (5.6)	4 (8.9)	(0.0)

*Surgery*				
MRM	20 (28.2)	—	20 (76.9)	—
MRM + reconstruction	5 (7.0)	—	5 (19.2)	
BCS + ALND	1 (1.4)	—	1 (3.8)	

DRT = definitive radiotherapy; NART = neoadjuvant radiotherapy; MRM = mastectomy; BCS = breast-conserving surgery; ALND = axillary lymph node dissection.

**Table 3:** Tumor stage and clinical/pathological-complete response after treatment.

Parameters			Total (*n* = 71)	DRT (*n* = 45)	NART (*n* = 26)	*p*
			No. (%)	No. (%)	No. (%)	
Pre-RT/post-NST	ycStage	0-II	8 (11.3)	8 (17.8)	0 (0.0)	0.007
		IIIA	5 (7.0)	3 (6.7)	2 (7.7)	
		IIIB	29 (40.8)	13 (28.9)	16 (61.5)	
		IIIC	20 (28.2)	12 (26.7)	8 (30.8)	
		IV	9 (12.7)	9 (20.0)	0 (0.0)	
	yc	ycT0	1 (1.4)	1 (2.2)	0 (0.0)	0.547
		ycN0	21 (29.6)	14 (31.1)	7 (26.9)	
		ycCR	2 (2.8)	2 (4.4)	0 (0.0)	

Post-RT	ycStage	0-II	35 (49.3)	21 (46.7)	14 (53.8)	0.021
		IIIA	6 (8.5)	2 (4.4)	4 (15.4)	
		IIIB	8 (11.3)	3 (6.7)	5 (19.2)	
		IIIC	11 (15.5)	8 (17.8)	3 (11.5)	
		IV	11 (15.5)	11 (24.4)	0 (0.0)	
	yc	ycT0	2 (2.8)	1 (2.2)	1 (3.8)	0.558
		ycN0	29 (40.8)	17 (37.8)	12 (46.2)	
		ycCR	11 (15.5)	9 (20.0)	2 (7.7)	

Post-surgery	ypStage	0-II	—	—	16 (61.5)	—
		IIIA	—	—	4 (15.4)	
		IIIB	—	—	3 (11.5)	
		IIIC	—	—	3 (11.5)	
	yp	ypT0	—	—	1 (3.8)	—
		ypN0	—	—	12 (46.2)	
		ypCR	—	—	5 (19.2)	

DRT = definitive radiotherapy; NART = neoadjuvant radiotherapy; RT = radiotherapy; NST = neoadjuvant systemic therapy; yc/ypCR = post-neoadjuvant therapy clinical/pathological-complete response.

**Table 4:** Univariate and multivariate analyses of patient clinical and treatment-related factors for total cCR and any cCR.

Parameters		Total cCR					Any cCR				
		Non-total cCR *N* (%)	cCR *N* (%)	UVA		MVA	Non-any cCR *N* (%)	Any cCR *N* (%)	UVA		MVA
				*χ * ^2^	*p*	*p*			*χ*2	*p*	*p*
Age	>40	44 (88.0)	6 (12.0)	1.58	0.209	0.546	23 (46.0)	27 (54.0)	1.86	0.173	0.405
	≤40	16 (76.2)	5 (23.8)				6 (28.6)	15 (71.4)			

Molecular subtype	Luminal A	5 (50)	5 (50)	14.98	0.002		1 (10.0)	9 (90.0)	10.90	0.012	
	Luminal B	11 (73.3)	4 (26.7)				5 (33.3)	10 (66.7)			
	Her2-overexpressed	14 (93.3)	1 (6.7)				4 (26.7)	11 (73.3)			
	Triple-negative	30 (96.8)	1 (3.2)				19 (61.3)	12 (38.7)			
	Other subtypes vs. Her2-overexpressed			1.132	0.287				1.582	0.208	
	Other subtypes vs. Triple-negative			6.324	0.012	0.033			9.520	0.002	0.003

TNM stage (pre-RT)	0-IIIA	9 (69.2)	4 (30.8)	2.84	0.092	0.183	4 (30.8)	9 (69.2)	0.67	0.414	0.730
	IIIB-IV	51 (87.9)	7 (12.1)				25 (43.1)	33 (56.9)			

Boost	No	26 (92.9)	2 (7.1)	2.46	0.117	0.420	14 (50.0)	14 (50.0)	1.60	0.205	0.713
	Yes	34 (79.1)	9 (20.9)				15 (34.9)	28 (65.1)			

Concurrent chemotherapy	No	24 (77.4)	7 (22.6)	2.11	0.146	0.723	10 (32.3)	21 (67.7)	1.68	0.195	0.791
	Yes	36 (90.0)	4 (10.0)				19 (47.5)	21 (52.5)			

RT = radiotherapy; cCR = clinical complete response; UVA = univariate analysis; MVA = multivariate analysis.

**Table 5:** Patterns of progression.

*Progression at any site*			
Parameters	Total (%) (*n* = 41)	DRT (%) (*n* = 31)	NART (%) (*n* = 10)
LRP alone	5 (12.2)	5 (16.1)	0 (0.0)
DP alone	15 (36.6)	9 (29.0)	6 (60.0)
LRP + DP	21 (51.2)	17 (54.8)	4 (40.0)

*Locoregional progression*			
Parameters	Total (%) (*n* = 26)	DRT (%) (*n* = 22)	NART (%) (*n* = 4)
Breast or CW alone	18 (69.2)	16 (72.7)	2 (50.0)
Regional nodes alone	5 (19.2)	3 (13.6)	2 (50.0)
Axilla alone	1	1	0
IMN alone	2	1	1
SCV/ICV alone	1	1	0
Multiple regions	1	0	1
CW + regional nodes	3 (11.5)	3 (13.6)	0 (0.0)

DRT = definitive radiotherapy; NART = neoadjuvant radiotherapy; LRP = locoregional progression; DP = distant progression; CW = chest wall; SCV = supraclavicular; ICV = infraclavicular; IMN = internal mammary nodes.

**Table 6:** Univariate and multivariate analyses of patient clinical and treatment-related factors for LRPFS and PFS.

Parameters	LRPFS			PFS		
	1-year LRPFS (%)	*p*		1-year PFS (%)	*p*	
		UVA	MVA		UVA	MVA
Age		0.033	0.222		0.272	0.576
≤40	94.7			66.7		
>40	72.5			54.8		
Menopausal status		0.057	0.465		0.121	0.428
Pre-menopause	84.1			60.3		
Post-menopause	73.0			54.0		
Pre-treatment TNM stage		0.261	0.058		0.315	0.182
IIIA + IIIB	79.9			57.6		
IIIC + IV	79.0			57.8		
Molecular subtype		0.006	0.268		0.003	0.061
Luminal A/B + HER2-overexpressed	91.4			74.0		
Triple-negative	62.4			37.6		
Boost		0.487	0.975		0.462	0.734
No	68.0			54.7		
Yes	85.3			60.3		
Concurrent chemotherapy		0.533	0.618		0.317	0.403
No	82.5			58.4		
Yes	76.4			57.8		
cCR		<0.001	<0.001		0.001	0.002
Non-any cCR	58.6			37.5		
Any cCR	92.2			72.1		

LRPFS = locoregional progression-free survival; PFS = progression-free survival; cCR = clinical complete response; UVA = univariate analysis; MVA = multivariate analysis.

## Data Availability

The datasets generated and/or analyzed during the current study are available from the corresponding author on reasonable request.
